# Soluble and nuclear oestrogen receptor status of advanced endometrial cancer in relation to subsequent clinical prognosis.

**DOI:** 10.1038/bjc.1987.111

**Published:** 1987-05

**Authors:** L. Castagnetta, M. Lo Casto, O. M. Granata, M. Calabro, M. Ciaccio, R. E. Leake

## Abstract

Both soluble and nuclear oestrogen receptors have been measured in at least two separate sections from 72 endometrial cancers and 12 normal endometria. Concentration of oestrogen receptor is shown to be, in our hands, more meaningful when expressed per unit DNA than per unit protein, whether for soluble or nuclear receptor. Endometrial cancer cells from the central part of the tumour are shown to be receptor negative more frequently than those from peripheral tumour. Thus, in large cancers, biopsies from different areas are required before a tumour can be correctly designated as receptor positive, heterogeneous or receptor negative. The intratumoral variation of receptor status may relate to poor prognosis, since patients with homogeneous receptor-positive disease survive significantly longer than those with tumours showing either heterogeneous distribution of receptor or homogeneous absence of receptor. Intratumoral variation in receptor status is found to be more common in the group of patients who are within 7 years of their menopause, than in older patients.


Br. J. Cancer (1987), 55, 543–546 © The Macmillan Press Ltd., 1987

### Soluble and nuclear oestrogen receptor status of advanced endometrial cancer in relation to subsequent clinical prognosis

L. Castagnetta[1]*, M. Lo Casto[1], O.M. Granata[1], M. Calabro[1], M. Ciaccio[1] & R.E. Leake[2]

[1]*Hormone Biochemistry Laboratory, Biochemistry Institute, Faculty of Medicine, University of Palermo, Policlinico 90127, Palermo, Italy; and* [2]*Department of Biochemistry, University of Glasgow, Glasgow G12 8QQ, UK.*

**Summary** Both soluble and nuclear oestrogen receptors have been measured in at least two separate sections from 72 endometrial cancers and 12 normal endometria. Concentration of oestrogen receptor is shown to be, in our hands, more meaningful when expressed per unit DNA than per unit protein, whether for soluble or nuclear receptor. Endometrial cancer cells from the central part of the tumour are shown to be receptor negative more frequently than those from peripheral tumour. Thus, in large cancers, biopsies from different areas are required before a tumour can be correctly designated as receptor positive, heterogeneous or receptor negative. The intratumoral variation of receptor status may relate to poor prognosis, since patients with homogeneous receptor-positive disease survive significantly longer than those with tumours showing either heterogeneous distribution of receptor or homogeneous absence of receptor. Intratumoral variation in receptor status is found to be more common in the group of patients who are within 7 years of their menopause, than in older patients.

In Sicily, the incidence of endometrial cancer is increasing. Standardized mortality rate, per 100,000 women, was 9.66 for endometrial cancer (Cislaghi *et al.*, 1978). However, this rate is thought to be an underestimate (Castagnetta *et al.*, 1980). Further, endometrial cancer clinics in Palermo have recently seen a rapid increase in new cases. For this reason, better indices of prognosis and therapy selection are required. The relative frequency of locally advanced disease in Sicily provides adequate tissue for an appropriate study.

The value of the steroid receptor status in the management of endometrial cancer has recently been reviewed (Soutter & Leake, 1987).

The first studies of oestrogen receptor content (in breast cancer) were confined to the soluble fraction (Jensen *et al.*, 1973; Mass *et al.*, 1975). However, a significant proportion of tumours may contain non-functional receptor in the soluble fraction (Laing *et al.*, 1977; Spelsberg & Boyd-Leinen, 1980). Improved prediction of response to endocrine therapy has been achieved through measuring nuclear oestrogen receptor (Barnes *et al.*, 1979; Hahnel *et al.*, 1980; Leake *et al.*, 1981a), progesterone receptor (Bloom *et al.*, 1980; Hawkins *et al.*, 1980; Osborne *et al.*, 1980) a product of oestrogen action, oestrogen-induced enzyme activity (Duffy & O'Connell, 1981) and oestrogen-induced protein secretion (Veith *et al.*, 1983). We have selected nuclear oestrogen receptor because this assay is relatively easy, oestrogen nuclear receptor is relatively stable and the amount of tissue needed is relatively small (Leake *et al.*, 1981a).

In this paper, we report the measurement of soluble and nuclear oestrogen receptor content of biopsies from both the centre and the edge of locally advanced endometrial cancers. Evidence is presented that the results are best expressed relative to the DNA content of the original suspension. Using this approach, we have examined the significance of intratumoral variation of receptor content in relation to the subsequent behaviour of the disease.

### Materials and methods

### *Patients*

Seventy-two patients were included in the study. All attended the Gynaecologic Clinics of the City Hospital, the University Hospital or the Cancer Hospital Centre, Palermo. Of the 72, 8 patients were pre-menopausal (mean age 44.6 yr), 19 were less than seven years post-menopausal (mean age 54.9) and 45 were more than 7 years post-menopausal (mean age 66.7). Menopause was taken as the time of the last menstrual bleed.

### *Tissue collection and storage*

Tissue was collected, on ice, fresh from the operating theatre. Adhering fat and obviously necrotic tissue was removed before adjacent sections were taken for pathological examination and oestrogen receptor assay. Separate sections were removed from the edge and the more central part of the tumour. Tissue was either processed fresh or stored at −20°C in sucrose-glycerol buffer [0.25 M sucrose, 10 mM HEPES, 1.5 mM $MgCl_2$, pH 7.4, 50% glycerol(v/v)]. Under these conditions of storage, concentration of both soluble and nuclear oestrogen receptor has been shown to be stable for at least three months in both breast and endometrial cancer biopsies (Leake *et al.*, 1979; Crawford *et al.*, 1984). The molecular form of the receptor, as determined by sucrose density gradient analysis, is also stable under these storage conditions for 60–90 days (Crawford *et al.*, 1984). Normal tissue was obtained from pathologically normal regions of hysterectomy samples.

### *Tissue fractionation*

Stored tissue was re-hydrated for 15 min at 4°C in sucrose buffer (0.25 M sucrose, 10 mM HEPES, 1.5 mM $MgCl_2$, pH 7.4). A section of approximately 150 mg tissue was homogenised in freshly-made HED buffer (20 mM HEPES, 1.5 mM EDTA, 0.25 mM Dithiothreitol, pH 7.4) at 50 mg ml$^{-1}$ for 2 × 10 sec bursts at a setting of 150 on an Ultra-Turrax model TP 18/2. The homogenate was examined under the phase-contrast microscope and further homogenised, as necessary, in a glass tissue grinder (Kontes Duall) until ~80% of the cells were lysed. At no stage was the temperature of the homogenate permitted to rise above 4°C. Normal tissue was adequately homogenised in a teflon-glass homogeniser. The homogenate was centrifuged at 5,000 *g* for 10 min at 4°C to yield a cytosol and a crude nuclear pellet. The pellet was washed three times in buffered saline (0.15 M NaCl, 10 mM HEPES, pH 7.4) and finally resuspended, using the Kontes grinder, to the original volume in buffered saline. Incorporation at this stage of a wash in 0.1% Triton X-100 did not significantly reduce the level of nuclear binding.

*Present address: Institute of Oncology School of Medicine, University of Messina, 98100 Messina, Italy.
Correspondence: R.E. Leake.
Received 5 January 1987.

Further purification of the nuclear pellet by differential centrifugation through sucrose (final layer was 2.4 M sucrose) did not alter receptor content per unit DNA.

*Receptor measurement*

Incubation of either the cytosol or nuclear pellet with $^3$H-oestradiol ($\pm$100-fold excess diethylstilboestrol as competitor) was carried out at seven concentrations in the range $10^{-10}-10^{-9}$ M. All tubes were incubated at 4°C for 18 h or 20°C for 2 h, both conditions give similar receptor content (Love *et al.*, 1983).

Unbound steroid was removed from the cytosol (DCC) and nuclear fractions (filtration through Whatman GF/C discs) as previously described (Leake, 1980). Results were analysed by the method of Scatchard using a simple computer programme (written for the Olivetti M20). Receptor content was only reported as positive if at least five points could be used to determine a 'best fit' line and if the dissociation constant ($K_d$) lay in the range $5\times10^{-11}-5\times10^{-10}$ M. Intra-assay variation was within 5% (six separate samples) and inter-assay variation within 12% (ten separate assays) using lyophilized quality control samples of human myometrium and endometrial cancer.

Protein and DNA content were determined by the methods of Lowry (Lowry *et al.*, 1951) and Burton (as modified by Katzenellenbogen and Leake, 1974), respectively.

### Results

*Expression of receptor concentration*

Quantitative data on receptor content of breast and endometrial tumours varies considerably between laboratories (Cowan & Leake, 1984). For soluble oestrogen receptor, this is, in part, due to the large variations in estimation of protein content of similar cytosols. Use of DNA content as a reference point for soluble oestrogen receptor content has been proposed since it is less sensitive to assay reagents such as dithiothreitol and molybdate. In normal endometrium, goodness of fit to a normal distribution of soluble oestrogen receptor was $P<0.001$ relative to protein but $0.5<P<0.75$ relative to DNA. Similar results were obtained with tumour tissue. Thus statistical comparison of receptor content of different groups of tissue is best made when receptor concentration is referred to the DNA content of the original homogenate.

*Cellularity and histology*

Grade I tumours contained 25–50% tumour cells. Less well differentiated tumours were more highly cellular, with grade III tumours containing 40–75% tumour cells. Within any one histological grade, tumours that were uniformly oestrogen receptor positive were associated with lower cellularity than those which had both receptor positive and receptor negative components. As reported previously (Castagnetta *et al.*, 1983), the proportion of grade III tumours is higher in Sicily than elsewhere.

*Receptor content*

The mean receptor content of the 56 receptor positive samples taken from the edge of each tumour is shown in Table I. Relative receptor content of a small group of histologically normal endometrium is also shown. The mean concentration of receptor in either soluble or nuclear fraction was very similar to that of normal secretory endometrium, whether expressed per unit protein or DNA. For review of the change in receptor content of the normal endometrium during the menstrual cycle (Soutter & Leake, 1987).

*Distribution of receptor*

For each patient, receptor status was determined in both the soluble and nuclear fractions of at least two separate parts of the tumour. Thus, an individual tumour was classified as (+ +) if *both* soluble and nuclear oestrogen receptor were present in *all* sections of the tumour examined. If receptor was missing from either soluble or nuclear fraction of one biopsy, then the tumour was (+ −) and if receptor was absent from all fractions, then the tumour was classified as (− −). Using this classification, receptor status of tumours from the 72 patients can be summarised as shown in Figure 1. The data indicate that loss of receptor from one fraction (+ −) is more common in the early post-menopausal group (7 out of 19; 37%) than in the late menopausal group (5 out of 45; 11%). This difference is statistically significant ($\chi^2$test: $0.05>P>0.025$).

*Intratumoral variations in receptor content*

Table II shows the mean data for all receptor positive biopsies taken from either the periphery or the more central (presumably older) part of the tumour for the 51 patients in the follow-up study (see later). The concentrations are similar to those previously reported for locally advanced endometrial cancer (Billiat *et al.*, 1982). Soluble receptor was found in 51% of biopsies from the central portion (26 out of 51) and in 67% from the periphery (34 out of 51). Although the incidence of receptor positive tissue was lower in the central portions, the concentration of receptor within receptor-positive cells from either the central or peripheral portions was statistically indistinguishable. The apparent increase in loss of receptor from the central portion is unlikely to be due to cell necrosis since (a) obviously necrotic areas were removed before assay, and (b) pathology of the adjacent section was checked carefully during the determination of cellularity.

*Relation of receptor distribution to clinical progress*

Only those patients for whom full FIGO classification (UICC-TNM, 1974) was possible, were included in the follow-up study. However, this sub-group of 51 patients was identical to the original 72 in terms of distribution between

**Table I** Oestrogen receptor content of normal secretory endometrium and endometrial cancer

| | | Cytosol | | Nuclear |
|---|---|---|---|---|
| | %ER+ | fmol mg$^{-1}$ Pr. | fmol µg$^{-1}$ DNA | fmol µg$^{-1}$ DNA |
| Normal $n=12$ | 100 | 68.1±45.3 | 2.1±1.0 | 1.9±1.4 |
| Endometrial cancer $n=72$ | 75 | 62.4±44.2 | 2.4±1.9 | 2.1±1.4 |

All values in the tables are expressed as Mean±S.D. The endometrial cancer values are the means of all oestrogen receptor positive samples taken from the peripheral portions of tumours.

**Figure 1** The distribution of patients among the three receptor status groups is shown relative to menstrual status. Group 1 ($n=8$) is pre-menopausal, Group 2 ($n=19$) is <7 yr post-menopausal and Group 3 ($n=45$) is >7 yr post-menopausal. Uniform receptor positive status (+ +) is indicated by □, variable status (+ −) by ▥ and uniform receptor negative status (− −) by ■.

**Table II** Mean concentration of soluble and nuclear oestrogen receptor in endometrial cancer biopsies in relation to menopausal status

| *Menopausal status* | *Cytosol* $fmol\,mg^{-1}$ *Pr.* | *Cytosol* $fmol\,\mu g^{-1}$ *DNA* | *Nuclear* $fmol\,\mu g^{-1}$ *DNA* |
|---|---|---|---|
| Pre | | | |
| C $n=2$ | 50.0±46.7 | 1.4±2.0 | 1.5±1.5 |
| P $n=3$ | 48.2±48.8 | 2.3±4.8 | 2.3±2.2 |
| Post <7 yr | | | |
| C $n=6$ | 110.8±92.3 | 3.3±4.6 | 2.0±1.6 |
| P $n=9$ | 56.3±43.1 | 1.1±1.0 | 1.5±1.3 |
| Post >7 yr | | | |
| C $n=18$ | 63.1±46.1 | 2.1±2.0 | 1.6±1.7 |
| P $n=22$ | 60.9±48.8 | 2.3±2.4 | 1.6±1.3 |

C = central or older portion of tumour. P = peripheral portion of tumour. Numbers of tumours ($n$) reflect the number of receptor positive biopsies in each category. This Table is confined to the 51 patients included in the follow-up study of whom 4 were pre-menopausal, 13 were post-menopausal <7 yr and 34 post-menopausal >7 yr.

the various receptor classes (49% (+ +) in both cases, 33% instead of 32% in the (− −) group and 17% instead of 18% in the (+ −) group). The division by menopausal status (4 pre-menopausal, 13 early post-menopausal and 34 more than 7 yr post-menopausal) was also similar, although the pre-menopausal group is now too small for further analysis.

The clinical follow-up of the 51 patients is shown in Table III. The mean follow-up time of those still alive was 55 months (range 36–69 mo.) The mean survival time for the 14 dead was 16.71 mo. One patient in the (+ +) group died from non-cancer-related disease (diabetic coma). Of those who have died from endometrial cancer, the mean survival

**Table III** Follow-up study of 51 adenocarcinoma patients having 55 months mean follow-up

| *Year* | *Total* | *HS Status* | *Alive* | *Dead* |
|---|---|---|---|---|
| 1980 | 12 | (+ +) | 9 | 3 |
| $n=23$ | 5 | (− −) | 3 | 2 |
| | 6 | (+ −) | 4 | 2 |
| 1981 | 9 | (+ +) | 7 | 2 |
| $n=18$ | 8 | (− −) | 6 | 2 |
| | 1 | (+ −) | 1 | 0 |
| 1982 | 4 | (+ +) | 3 | (1*) |
| $n=10$ | 4 | (− −) | 3 | 1 |
| | 2 | (+ −) | 1 | 1 |

*This patient died from a diabetic coma. Year indicates year of presentation.

time for the five patients with (+ +) tumours was 26.0±11.5 mo., whereas that for the three in the (+ −) group was 8.33±2.5 mo. The two groups appeared equivalent in terms of histological grade (almost all grade II) and extent of myometrial invasion.

### Discussion

The accepted method for expression of soluble receptor content is relative to protein content of the tissue. Even in normal endometrium however, this leads to an abnormal or skewed distribution of the data. For this reason, receptor values may be more comparable when expressed relative to DNA content of the original suspension, even if only soluble receptor content is being determined.

In larger tumours, not only can receptor content vary across a tumour (Leake *et al.*, 1979) but even receptor status can vary from positive to negative across individual tumours (Figure 1). The follow-up of the patients (Table III) indicates the possible clinical significance of a heterogenous receptor distribution across a tumour. Clinically, it is not easy to determine whether an advanced local tumour is slow growing or aggressive. Multiple biopsies of such a tumour for determination of functional receptor status may be useful. Those tumours which are receptor positive throughout, reflect a relatively good prognosis, whereas variation in receptor status across the tumour may be associated with poor prognosis. In conclusion, assay of functional receptors (measured as soluble and tightly bound receptor in the homogenate) from different parts of large endometrial cancers may be a measure of both hormone sensitivity and prognosis.

Antibodies to oestrogen receptor have the potential to reveal the extent of heterogeneity of receptor distribution in any tumour (Crawford *et al.*, 1985; King *et al.*, 1985). However, until their value has been fully assessed, the biochemical approach, described here, remains the best way to detect heterogeneous tumours. The optimal therapy for tumours heterogeneous in receptor status may well differ from that for homogeneous receptor positive tumour in that some combination or, rather, sequence of endocrine and chemotherapy may be more appropriate for the former type of tumour.

This study was part of a special project on Control of Neoplasic Growth assisted by C.N.R. contract no. 83.0765.96 (L.C.).

We are pleased to acknowledge essential financial support from the Cancer Research Campaign (R.E.L.) and the CIBA Foundation (L.C.) as well as Professor F. Cacioppo for his suggestions, criticism and advice.

The authors also thank the Ing. C. Olivetti & C. for their generous gift of a computer (M20).

### References

BARNES, D.M., SKINNER, L.G. & RIBEIRO, G.G. (1979). Triple hormone-receptor assay: A more accurate predictive tool for the treatment of advanced breast cancer. *Br. J. Cancer*, **40**, 682.

BILLIAT, G. DE HERTOGH, R., BONTE, J., IDE, P. & VLAEMYNCK, G. (1982). Estrogen receptors in human uterine adenocarcinoma: Correlation with tissue differentiation, vaginal karypycnotic index, and effect of progestogen or anti-estrogen treatment. *Gynecol. Oncol.*, **14**, 33.

BLOOM, N.D., TOBIN, E.H., SCHREIBMAN, B. & DEGENSHEIN, G.A. (1980). The role of progesterone receptors in the management of advanced breast cancer. *Cancer*, **45**, 2992.

CASTAGNETTA, L., COICO, L., GRANATA, O.M., RUSSELLO, T., SAVAROLA, L.R., SPINA, G., TRAINA, A. & DARDANONI, L. (1980). A study of distortion of cancer death data in Palermo. *L'Igiene Moderna*, **74**, 248.

CASTAGNETTA, L., LO CASTO, M., MERCANDANTE, T., POLITO, L., COWAN, S. & LEAKE, R.E. (1983). Intratumoral variation of oestrogen receptor status in endometrial cancer. *Br. J. Cancer*, **47**, 261.

CISLAGHI, C., DE CARLI, A., MOROSINI, P. & PUNTONI, R. (1978). Atlante della mortalita per tumori in Italia. Trienno 1970–72. *Lega Italianna per la lotta contro i tumori*, Roma.

COWAN, S.K. & LEAKE, R.E. (1984). Quality control of steroid receptor assays in breast cancer. The British experience. In *Clinical interest of steroid hormone receptors in breast cancer: European experience*, Leclercq, G. *et al.* (eds) p. 98, Springer-Verlag, Berlin.

CRAWFORD, D., COWAN, S., HYDER, S., McMENAMIN, M., SMITH, D. & LEAKE, R. (1984). A new storage procedure for tumor biopsies prior to estrogen receptor measurement. *Cancer Res.*, **46**, 2348.

CRAWFORD, D.J., LOPE-PIHIE, A., COWAN, S., GEORGE, W.D. & LEAKE, R.E. (1985). Pre-operative determination of oestrogen receptor status in breast cancer by immunocytochemical staining of fine needle aspirates. *Br. J. Surg.*, **72**, 991.

DUFFY, M.J. & O'CONNELL, M. (1981). Estrogens, estradiol receptors and peroxidase activity in human mammary carcinomas. *Eur. J. Cancer*, **17**, 711.

HAHNEL, P., PARTIDGE, R.R., GAVEL, L., TWADDLE, E. & RABDIZAL, T. (1980). Nuclear and cytoplasmic estrogen receptors and progesterone receptors in breast cancer. *Eur. J. Cancer*, **16**, 1027.

HAWKINS, R.A., ROBERTS, M.M. & FORREST, A.P.M. (1980). Oestrogen receptors and breast cancer: Current status. *Br. J. Surg.*, **6**, 153.

JENSEN, E.V., BLOCK, G.E., SMITH, S. & DESOMBRE, E.R. (1973). Hormone dependence of breast cancer. *Rec. Res. Cancer Res.*, **42**, 55.

KATZENELLENBOGEN, B.S. & LEAKE, R.E. (1974). Distribution of the oestrogen-induced protein and of total protein between endometrial and myometrial fractions of the immature and mature rat uterus. *J. Endocrinol.*, **63**, 439.

KING, W.J., DESOMBRE, E.R., JENSEN, E.V. & GREENE, G.L. (1985). Comparison of immunocytochemical and steroid binding assays for estrogen receptor in human breast tumors. *Cancer Res.*, **45**, 293.

LAING, L.M., SMITH, D.C., CALMAN, K.C., SMITH, M.G. & LEAKE, R.E. (1977). Nuclear estrogen receptors and treatment of breast cancer. *Lancet*, **ii**, 168.

LEAKE, R.E. (1980). Methodology of steroid hormone determination in breast cancer. In *Progestogens in the management of hormone responsive carcinomas*, Taylor, R.W. (ed) p. 3. Medicine, Oxford.

LEAKE, R.E., LAING, L. & SMITH, D.C. (1979). A role for nuclear oestrogen receptors in prediction of therapy regime for breast cancer patients. In *Steroid receptor assays in human breast tumours: Methodological and clinical aspects*, King, R.J.B. (ed) p. 73. Alpha Omega, Cardiff.

LEAKE, R.E., LAING, L., CALMAN, K.C., MACBETH, F.R., CRAWFORD, D. & SMITH, D.C. (1981a). Oestrogen receptor status and endocrine therapy of breast cancer: Response rates and status stability. *Br. J. Cancer*, **43**, 59.

LOVE, C.A., COWAN, S.K., LAING, L.M. & LEAKE, R.E. (1983). Stability of human nuclear oestrogen-receptor complex: Influence of temperature and ionic strength. *J. Endocrinol.*, **99**, 423.

LOWRY, O.H., ROSEBROUGH, N.J., FARR, A.L. & RANDALL, R.J. (1951). Protein measurement with the Folin-phenol reagent. *J. Biol. Chem.*, **193**, 265.

MASS, H., ENGEL, B., TRAMS, G., NOWAKOWSKI, H. & STOLZENBACH, G. (1975). Steroid hormone receptors in human breast cancer and the clinical significance. *J. Steroid Biochem.*, **6**, 743.

OSBORNE, C.N., YOCHMOUNTS, M.G., KNIGHT, W.A. & McGUIRE, W.L. (1980). The value of estrogen and progesterone receptors in the treatment of breast cancer. *Cancer*, **46**, 2884.

SOUTTER, W.P. & LEAKE, R.E. (1987). Steroid receptors in gynaecological cancers. In *Advances in Obstetrics and Gynaecology* **15**. Bonnar, J. (ed). Churchill Livingstone, Edinburgh.

SPELSBERG, T.C. & BOYD-LEINEN, R.H. (1980). Identification of biologically active steroid receptors. *Clin. Biochem.*, **5**, 198.

UICC-TNM. (1974). Classification of malignant tumours. p. 61. IUAC Publications, Geneva.

VEITH, F.O., CAPONY, F., GARCIA, M. & 5 others (1983). Release of oestrogen induced glycoprotein with a molecular weight of 52,000 by breast cancer cells in primary culture. *Cancer Res.*, **43**, 1861.


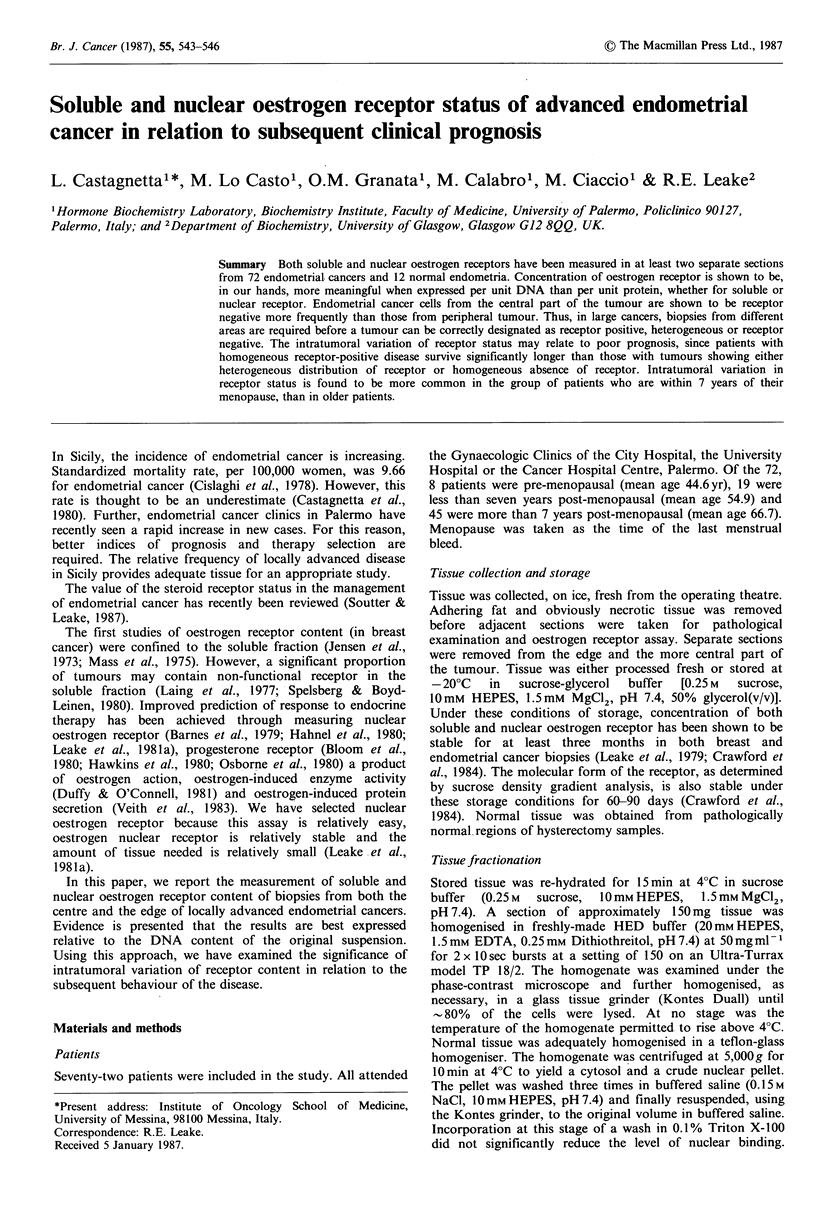

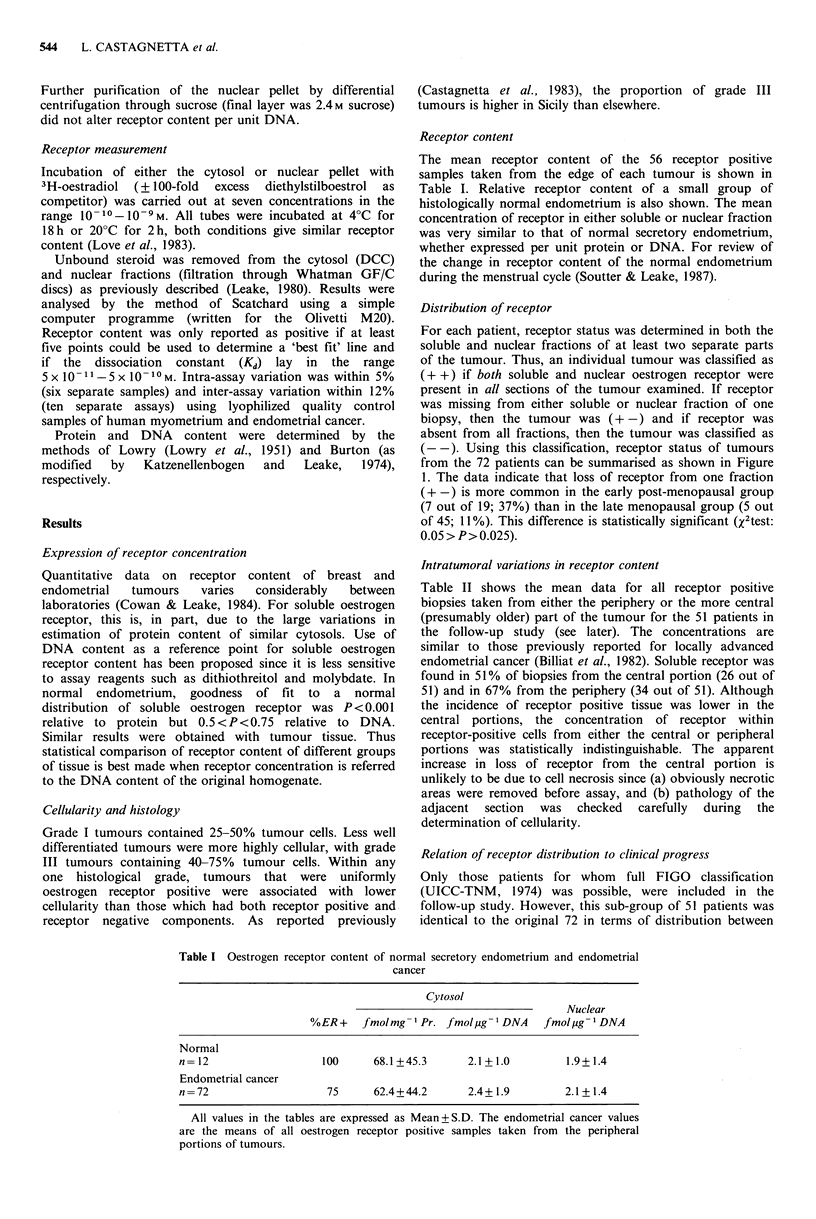

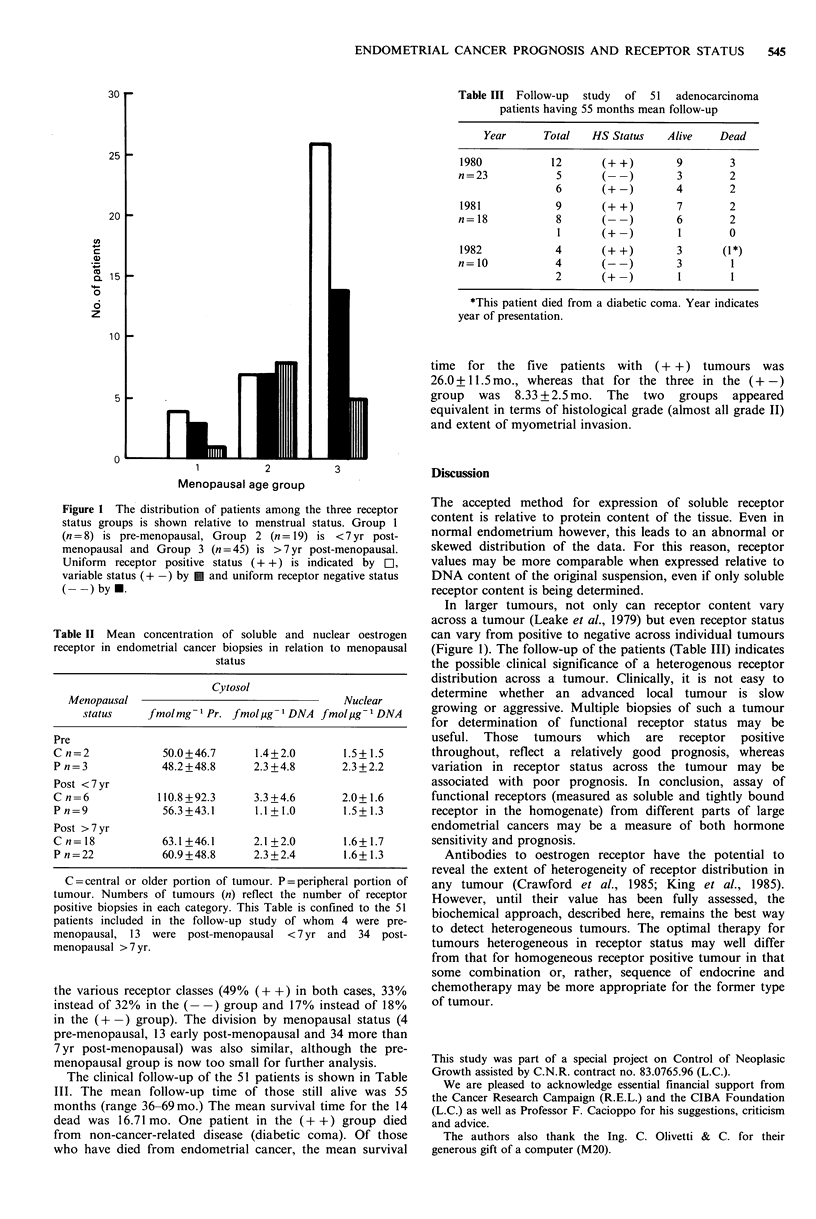

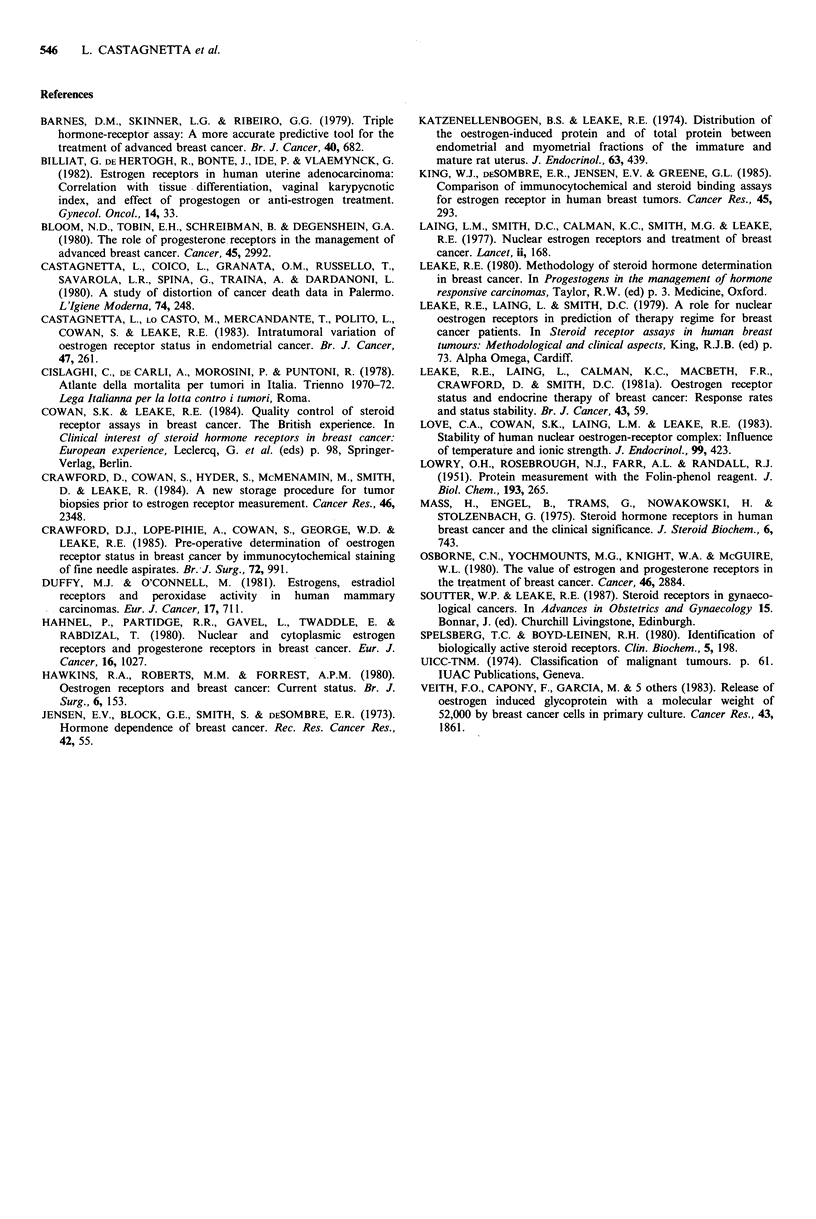


## References

[OCR_00446] Bloom N. D., Tobin E. H., Schreibman B., Degenshein G. A. (1980). The role of progesterone receptors in the management of advanced breast cancer.. Cancer.

[OCR_00459] Castagnetta L., Lo Casto M., Mercadante T., Polito L., Cowan S., Leake R. E. (1983). Intra-tumoural variation of oestrogen receptor status in endometrial cancer.. Br J Cancer.

[OCR_00481] Crawford D. J., Lope-Pihie A., Cowan S., George W. D., Leake R. E. (1985). Pre-operative determination of oestrogen receptor status in breast cancer by immunocytochemical staining of fine needle aspirates.. Br J Surg.

[OCR_00475] Crawford D., Cowan S., Hyder S., McMenamin M., Smith D., Leake R. (1984). New storage procedure for human tumor biopsies prior to estrogen receptor measurement.. Cancer Res.

[OCR_00487] Duffy M. J., O'Connell M. (1981). Estrogens, estradiol receptors and peroxidase activity in human mammary carcinomas.. Eur J Cancer.

[OCR_00498] Hawkins R. A., Roberts M. M., Forrest A. P. (1980). Oestrogen receptors and breast cancer: current status.. Br J Surg.

[OCR_00492] Hähnel R., Partridge R. K., Gavet L., Twaddle E., Ratajczak T. (1980). Nuclear and cytoplasmic estrogen receptors and progesterone receptors in breast cancer.. Eur J Cancer.

[OCR_00508] Katzenellenbogen B. S., Leake R. E. (1974). Distribution of the oestrogen-induced protein and of total protein between endometrial and myometrial fractions of the immature and mature rat uterus.. J Endocrinol.

[OCR_00514] King W. J., DeSombre E. R., Jensen E. V., Greene G. L. (1985). Comparison of immunocytochemical and steroid-binding assays for estrogen receptor in human breast tumors.. Cancer Res.

[OCR_00548] LOWRY O. H., ROSEBROUGH N. J., FARR A. L., RANDALL R. J. (1951). Protein measurement with the Folin phenol reagent.. J Biol Chem.

[OCR_00522] Laing L., Smith M. G., Calman K. C., Smith D. C., Leake R. E. (1977). Nuclear oestrogen receptors and treatment of breast cancer.. Lancet.

[OCR_00537] Leake R. E., Laing L., Calman K. C., Macbeth F. R., Crawford D., Smith D. C. (1981). Oestrogen-receptor status and endocrine therapy of breast cancer: response rates and status stability.. Br J Cancer.

[OCR_00543] Love C. A., Cowan S. K., Laing L. M., Leake R. E. (1983). Stability of the human nuclear oestrogen receptor: influence of temperature and ionic strength.. J Endocrinol.

[OCR_00553] Maass H., Engel B., Trams G. (1975). Steroid hormone receptors in human breast cancer and the clinical significance.. J Steroid Biochem.

[OCR_00559] Osborne C. K., Yochmowitz M. G., Knight W. A., McGuire W. L. (1980). The value of estrogen and progesterone receptors in the treatment of breast cancer.. Cancer.

[OCR_00569] Spelsberg T. C., Boyd-Leinen P. A. (1980). Identification of biologically active and inactive steroid receptors.. Clin Biochem.

[OCR_00577] Veith F. O., Capony F., Garcia M., Chantelard J., Pujol H., Veith F., Zajdela A., Rochefort H. (1983). Release of estrogen-induced glycoprotein with a molecular weight of 52,000 by breast cancer cells in primary culture.. Cancer Res.

